# Ephrin B3 interacts with multiple EphA receptors and drives migration and invasion in non-small cell lung cancer

**DOI:** 10.18632/oncotarget.11219

**Published:** 2016-08-11

**Authors:** Ghazal Efazat, Metka Novak, Vitaliy O. Kaminskyy, Luigi De Petris, Lena Kanter, Therese Juntti, Per Bergman, Boris Zhivotovsky, Rolf Lewensohn, Petra Hååg, Kristina Viktorsson

**Affiliations:** ^1^ Karolinska Biomics Center, Department of Oncology-Pathology, Karolinska Institutet, SE-171 76 Stockholm, Sweden; ^2^ Institute of Environmental Medicine, Division of Toxicology, Karolinska Institutet, SE-171 77 Stockholm, Sweden; ^3^ Department of Molecular Medicine and Surgery (MMK), Thoracic Surgery, Karolinska Institutet, SE-171 76 Stockholm, Sweden

**Keywords:** Ephrin B3, EphA2, NSCLC, migration, invasion

## Abstract

Ephrin receptors (Ephs) are reported to control metastatic signaling of non-small cell lung cancer (NSCLC) and other tumors. Here we show for the first time that blocking expression of the Eph ligand Ephrin B3 inhibits NSCLC cell migration and invasion. We demonstrate that Ephrin B3 directly binds the EphAs EphA2, EphA3, EphA4, and EphA5. EphA2 Ser897 was previously shown to drive migration propensity of tumor cells and our study reveals that EphA2 stays phosphorylated on Ser897 in the Ephrin B3/EphA2 complex in NSCLC cells of different histology. Moreover, we report that within such Ephrin B3/EphA2 complex both Akt Ser 129 and p38MAPK are found indicating a potential to drive migration/proliferation. We also found the EMT marker E-cadherin expression to be maintained or increased upon Ephrin B3 blockade in NSCLC cells. Expression of Ephrin B3 was furthermore analyzed in a cohort of NSCLC stage IA-IB cases (n=200) alongside EphA2 and Ephrin A1. We found that Ephrin B3 was concomitantly expressed with EphA2 and Ephrin A1 with higher Ephrin B3 levels found in non-squamous than in squamous tumors, whereas EphA2 was higher expressed in well-differentiated than in low-differentiated tumors. In the entire NSCLC cohort, Ephrin B3 expression was not linked to patient survival, whereas a high EphA2 expression was associated with improved survival (p=0.03). In conclusion, we show that blocking Ephrin B3 expression inhibits NSCLC proliferation-, migration- and invasion capacity which calls for further studies on interference with Ephrin B3 as a possible therapeutic avenue in this tumor malignancy.

## INTRODUCTION

Non-small cell lung cancer (NSCLC) is one of the top ranked tumor types when it comes to both incidence and cancer-associated death worldwide [1]. In more than 60% of the cases the NSCLC patients present with a metastatic disease and in this setting the 5-year survival rate is as low as 10% [2]. Thus there is a great medical need to find biomarkers and novel therapeutic approaches for this tumor type.

In normal cells, the Ephrin ligand-Ephrin receptor (Eph) growth factor signaling axis controls multiple functions including cell positioning and migration capacity (reviewed in [3]). Binding of the cell bound Ephrin ligands, which can be of A or B subtype, to the ligand binding domain of the Eph receptor, which also may be of A or B subtype, results in phosphorylation of tyrosine residues in the Eph kinase domain. This leads to subsequent signaling alteration in downstream kinase networks e.g. MAPKs, PI3K/Akt and Src, a signaling event called “forward signaling” (reviewed in [3]). Ephrin and Eph association may also trigger signaling in the ligand-expressing cell, a feature known as “reversed signaling” [3]. Deregulation of Eph signaling is common in tumors and may be a result of altered ligand or Eph subtype expression in tumor-or associated stroma, or may occur as a consequence of mutations within the Eph affecting either the ligand binding domain and/or the kinase pocket (reviewed in [4]). Also in NSCLC, multiple Ephrin/Ephs are known to have altered expression and/or function e.g. EphA2, EphA3, EphA4, EphA5 and EphB3 [5–11]. Thus global next generation sequencing has identified mutations in EphA3 and EphA5 in lung adenocarcinomas confined to both ligand binding- and kinase domains [7, 8]. The EphA2 has been reported to have a higher expression in NSCLC tumors than in normal surrounding non-tumor tissue in a large fraction of cases and EphA2 expression has been associated with poor prognosis/risk for metastasis in this tumor malignancy [5, 6]. EphA2 expression has also been associated with K-*Ras* mutations and EGFR activation but not with *EGFR* mutations [5, 6]. Recent findings also points towards a role of EphA2 in driving resistance towards inhibitors of mutated EGFR illustrating the importance of EphA2 in NSCLC malignancy also in response to clinically applied targeted therapy [31].

On molecular level, EphA2 has been shown to drive proliferation and invasion in multiple tumor forms including NSCLC, prostate cancer, and glioma [5, 6, 12–17]. Furthermore, binding of Ephrin A1 to EphA2 has in prostate cancer and glioma cells been demonstrated to block proliferation- and invasion signaling mediated by EphA2, an effect in part caused by inhibition of EphA2 Ser897 phosphorylation [15].

We previously reported that blocking Ephrin B3 expression sensitizes NSCLC cells to radiotherapy (RT) [18]. Phosphoproteomic profiling of NSCLC cells in which Ephrin B3 expression was inhibited revealed lack of both EphA2 Ser897 and Akt Ser129 phosphorylations, indicating a signaling interaction between Ephrin B3 and EphA2 [19]. Following these results, we here demonstrate that Ephrin B3 and EphA2 are concomitantly expressed in NSCLC cells of different histology and that blocking Ephrin B3 expression inhibits cell proliferation, migration and invasion *in vitro.* By immunoprecipitation and proximity ligation assay (PLA) *in situ* we for the first time show that Ephrin B3 interacts with EphA2 and other EphAs e.g. EphA3, EphA4 and EphA5. We also found that Ser897 phosphorylated EphA2 bound to Ephrin B3 is in complex with p38MAPK, phosphorylated Akt Ser129 and in some NSCLC cells also Src. Analyses of Ephrin B3 expression in stage IA-IB NSCLC clinical specimen revealed a concomitant expression with EphA2 and Ephrin A1 with higher Ephrin B3 expression in non-squamous than in squamous tumors. Our results did not reveal a link between high Ephrin B3 expression and poor patient survival whereas a high EphA2 expression was associated with improved survival (p=0.03) in this cohort of early stage NSCLC. In conclusion, we show that blocking Ephrin B3 expression inhibits NSCLC proliferation, migration and invasion capacity, which put forward studies on interference with Ephrin B3 signaling for possible therapeutic avenues in NSCLC.

## RESULTS

### Ephrin B3 regulates NSCLC cell proliferation, migration and invasion potential

**Figure 1:**
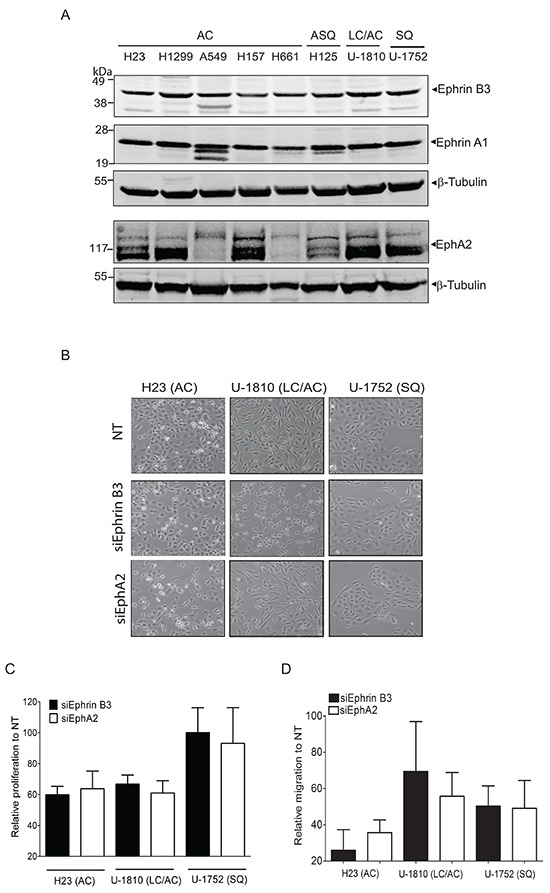
Endogenous expression of Ephrin B3 or EphA2 drives proliferation and migration of NSCLC cells **A.** NSCLC cell lines with the histology and omit the histologies comming afterwards histology adenocarcinoma (AC), adenosquamous carcinoma (ASQ), mixed phenotype (large cell/adenocarcinoma, LC/AC) or squamous (SQ) were profiled Ephrin B3, Ephrin A1 and EphA2 expression, using western blotting. To control equal loading among the samples, β-Tubulin was used. **B-D.** H23 (AC), U-1810 (LC/AC) or U-1752 (SQ) were treated with Ephrin B3-, EphA2- or non-targeting (NT) siRNAs for 24 h followed by either 24h-48h post incubation (24 h: H23, 48h: U-1810 and U-1752). The effects of Ephrin B3 or EphA2 siRNA knockdown on morphology **(B)**, proliferation **(C)**, migration **(D)** were analyzed. **(B)** Photos showing effects on cell morphology in H23 (AC), U-1810 (LC/AC) or U-1752 (SQ). (**C**) Cell proliferation was quantified by counting living H23 (AC), U-1810 (LC/AC) or U-1752 (SQ) cells, by Trypan blue. Presented results are the mean ± SD of three experiments. **(D)** Migration capacity was analyzed in H23, U-1810 and U-1752 at 48h post siRNA removal, after 24h of serum starvation and after 20h post seeding in a transwell assay. The number of cells on the membrane in the inserts were stained and counted. Data is given as % of migrating cells relative to NT obtained in three biological replicates and bars represent SD.

We previously showed that ablation of Ephrin B3 expression in NSCLC cells inhibits EphA2 Ser897 phosphorylation, suggesting a functional connection between Ephrin B3 and EphA2 [19]. To further understand the function of Ephrin B3 and associated EphAs in NSCLC, we profiled their expression levels in NSCLC cell lines of different histology (Figure 1). Ephrin B3 was homogenously expressed in all cell lines and EphA2 highly expressed in six out of the eight cell lines examined (Figure 1A). Ephrin A1, a *bona fide* ligand of EphA2 was indeed prominently expressed within the NSCLC cell line panel with no evident variation in expression (Figure 1A). We also found EphA3, EphA4 and EphA5 to be expressed in all NSCLC cell lines analyzed yet with less expression magnitude than EphA2 (Supplementary Figure S1).

*In vitro* studies have demonstrated that EphA2 regulates gliomas, prostate tumor cell and also NSCLC cell migration [6, 12–16, 17]. Forced overexpression of Ephrin B3 was similarly reported to increase invasion of glioma cells *in vitro* via a pathway involving Rac [20]. Here we examined if inhibition of Ephrin B3 could decrease proliferation, migration and invasion capacity of NSCLC cells using Ephrin B3 siRNA and blocked EphA2 for comparison (Figure 1B-D). Real time quantitative PCR of the NSCLC cells transfected with either Ephrin B3 or EphA2 siRNAs revealed 40-75% reduction in expression (Supplementary Figure S2). Blockade of Ephrin B3 or EphA2 expression altered morphology of both adenocarcinoma (AC) H23 cells and large cell/adenocarcinoma (LC/AC) U-1810 cells while no effect was seen in squamous cell (SQ) U-1752 (Figure 1B). In both AC H23 and LC/AC U-1810, knock-down of Ephrin B3 or EphA2 resulted in an about 40% inhibition of proliferation (Figure 1C). In contrast, in SQ U-1752 neither inhibition of Ephrin B3 nor EphA2 reduced proliferation capacity (Figure 1C). Importantly, Ephrin B3 knock-down reduced migration capacity by about 75% in AC H23 cells and by about 50% in SQ U-1752 cells respectively (Figure 1D, *black bars*). In LC/AC U-1810 a clear inhibition of migration was seen in two out of three biological replicates resulting in a mean inhibition of approximately 30% (Figure 1D, *black bars*). In line with previous reports [15], inhibition of EphA2 expression reduced migration with about 40-60% in the different NSCLC cell lines examined (Figure 1D, *white bars*). To address if Ephrin B3 influenced invasion capacity, the AC CL1-5 cell line previously reported to be invasive [21, 22] was used. The CL1-5 cells were confirmed to express Ephrin B3 (Supplementary Figure S3A) and were silenced for either Ephrin B3 or EphA2 expression by siRNA which resulted in an about 85% decrease in either Ephrin B3 or EphA2 mRNA expression (data not shown). Silencing of Ephrin B3 or EphA2 expression altered morphology of the CL1-5 cells (Supplementary Figure S3B). A decrease in proliferation by 60% and 40 % were also seen in the same cells upon Ephrin B3 or EphA2 siRNA treatment respectively (Supplementary Figure S3C). Under serum starvation, which diminished proliferation, blocking either Ephrin B3 or EphA2 expression decreased invasion with 90% and 80% respectively relative to Non-targeting (NT) siRNA treated CL1-5 cells (Supplementary Figure S3C). Thus our results demonstrate that inhibition of Ephrin B3 decrease NSCLC cell proliferation, migration and invasion and in line with previous reports corroborate a role of EphA2 blockade in the same signaling events.

### Silencing of Ephrin B3 causes increased expression of the epithelial to mesenchymal transition (EMT) signaling protein E-cadherin in some but not all NSCLC cells

**Figure 2:**
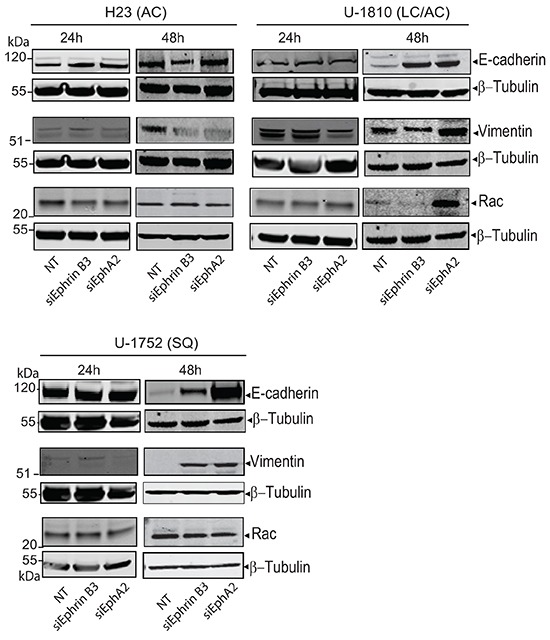
Down regulation of Ephrin B3 and EphA2 in NSCLC alter EMT marker expression H23 (AC), U-1810 (LC/AC) and U-1752 (SQ) cells were treated with Ephrin B3-, EphA2- or non-targeting (NT) siRNA. Indicated proteins were examined by western blot at 24h or 48h post siRNA addition. To control equal loading among the samples β-Tubulin was used.

EMT signaling controls migration and invasion capacity of tumor cells. Two of the hallmarks of tumor cells which have undergone EMT are the downregulation of the epithelial surface protein E-cadherin which control cell-cell contact and increase in vimentin expression (reviewed in [23]). Growth factor signaling is clearly linked to EMT and also Eph is reported to be influenced by EMT as well as to regulate it [23]. Thus EphA2 localization in breast cancer cells has been found to be controlled by E-cadherin [24] and Ephrin B3 was reported to influence EMT via the small GTPase Rac in glioma cells [20]. Given these reports on a link between Ephrin/Eph signaling and EMT markers in tumor cells we next analyzed E-cadherin, vimentin and Rac expressions upon knockdown of Ephrin B3 or EphA2 in NSCLC cells (Figure 2). AC H23 cells expressed prominent amounts of E-cadherin and no alteration in expression was evident after knockdown of either Ephrin B3 or EphA2 expression (Figure 2, *upper left panel*). In LC/AC U-1810 or SQ U-1752 which displayed low basal levels of E-cadherin, knock-down of Ephrin B3 or EphA2 increased E-cadherin expression at 48 h (Figure 2, *upper* *right panel and bottom panel*). Analysis of vimentin in AC H23 cells after either Ephrin B3- or EphA2 ablation showed decreased expression at 48 h post siRNA transfection (Figure 2, *upper right* *panel*). In LC/AC U-1810 cells only knockdown of Ephrin B3 reduced vimentin expression at 48h post siRNA transfection and in SQ U-1752 cells, vimentin expression in NT siRNA treated cells was low but increased after Ephrin B3 or EphA2 blockade (Figure 2, *bottom panel*). Rac was expressed in all three cell lines at basal level (Figure 2). In AC H23 or SQ U-1752 cells, manipulation of Ephrin B3 or EphA2 expression did not cause any major alteration in its expression while in LC/AC U-1810 in response to EphA2 but not Ephrin B3 siRNA an increased Rac level was evident.

### Ephrin B3 is a ligand of multiple EphA receptors and is in complex with EphA2 Ser897 in NSCLC cells

**Figure 3:**
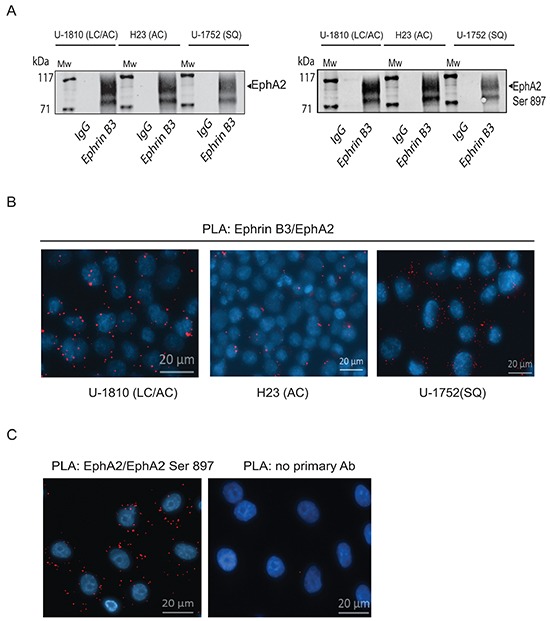

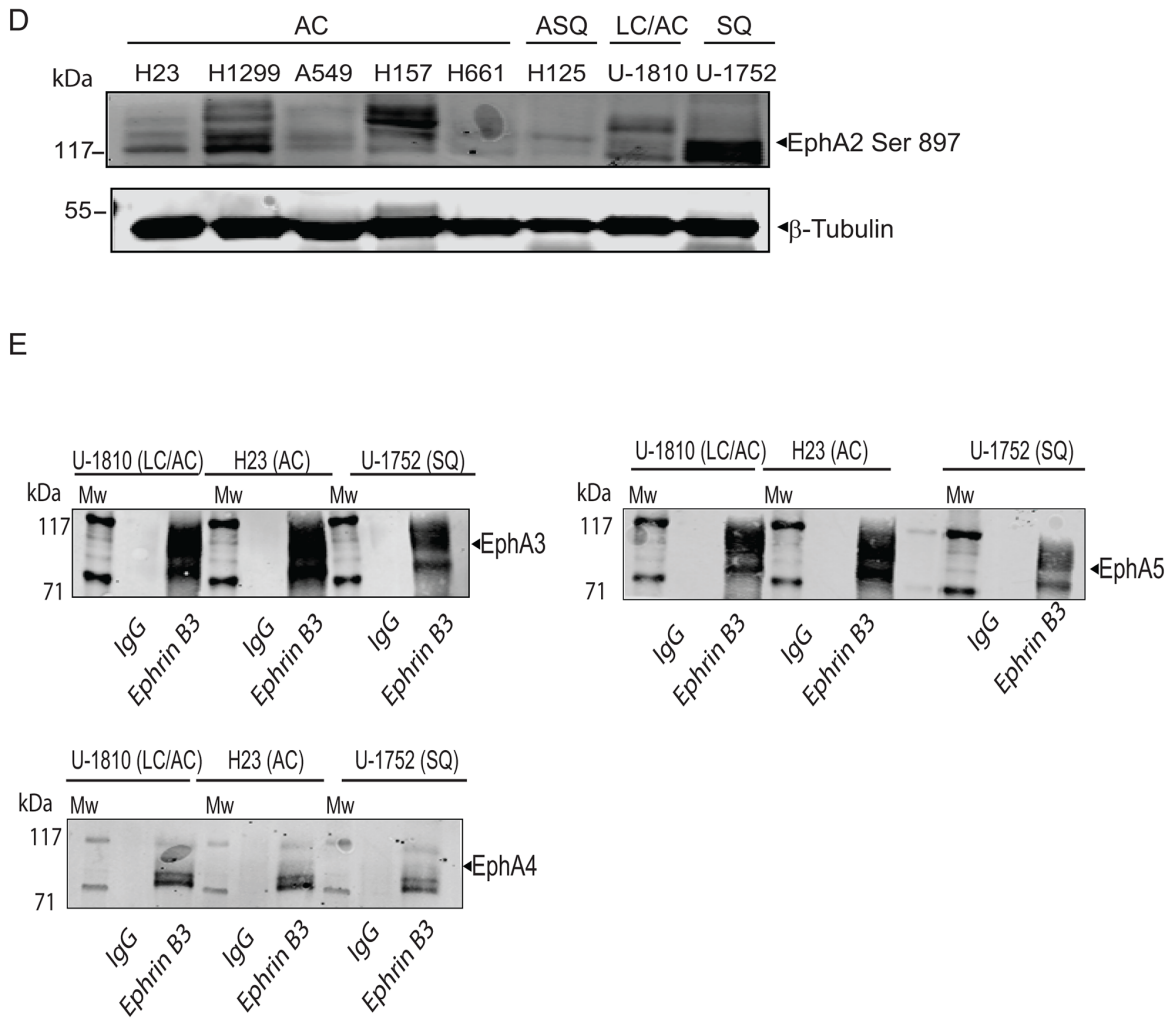
Ephrin B3 is a ligand of multiple EphA receptors The interactions of Ephrin B3 with Ephs were studied by immunoprecipitation in untreated NSCLC cells as indicated. **A.** Ephrin B3was immunoprecipitated from lysates of U-1810, H23, and U-1752 cells and the resulting immunocomplexes studied in western blot by antibodies against total EphA2 or EphA2 Ser897. **B.** The interaction between Ephrin B3 and EphA2 were analyzed by PLA *in situ* in untreated U-1810, H23 or U1-752 cells. Slides were incubated with Ephrin B3 and EphA2 antibodies and probed with Texas red (red) PLA probes with DAPI (blue) used for counterstaining cell nuclei. **C.** The interaction between total EphA2 and Ser897 phosphorylated EphA2 was verified with PLA in U-1810 cells. Texas-red labelled PLA probes were added (red) with DAPI (blue) used for counterstaining of nuclei. As a control U-1810 cells were used in PLA reactions omitting the primary antibodies. **D.** NSCLC cell lines were profiled for the expression of EphA2 Ser897, using western blotting. To control equal loading among the samples, β-Tubulin was used. **E.** The interactions of Ephrin B3 with Ephs were studied by immunoprecipitation in untreated NSCLC cells as indicated. Ephrin B3was immunoprecipitated from cell lysates of U-1810, H23, U-1810 and U-1752 and the resulting immunocomplexes analyzed by western blot with antibodies against EphA3, EphA4 and EphA5.

Ephrin A1 binding to EphA2 causes dephosphorylation of EphA2 Ser897 and reduces migration capacity in glioma- and prostate cancer cells [15]. Given the observed inhibition of NSCLC cell migration after blocking Ephrin B3 expression we next analyzed if Ephrin B3 directly binds to EphA2 and other Ephs in these cells (Figure 3).

By immunoprecipitation we indeed found that Ephrin B3 binds to EphA2 in NSCLC cells (Figure 3A, *left panel*). The interactions between Ephrin B3 and EphA2 in NSCLC cells were also validated by proximity ligation assay (PLA) *in situ* (Figure 3B). Ephrin B3 and EphA2 showed a clear association as indicated by the PLA signals, albeit different magnitudes of interaction were seen among the analyzed NSCLC cell lines (Figure 3B). Interestingly, immunoprecipitation of Ephrin B3 also pulled down Ser897- phosphorylated EphA2 (Figure 3A, *right panel*), indicating that Ephrin B3 may indeed constitute an EphA2 ligand that can enable EphA2 to stay phosphorylated on this site. Accordingly PLA confirmed Ser897 phosphorylation in NSCLC U-1810 cells (Figure 3C). Moreover, western blot analyses revealed prominent EphA2 Ser897 in several NSCLC cell lines indicating the existence of a functional Ephrin B3/EphA2 signaling loop in NSCLC cells (Figure 3D).

Immunoprecipitations also demonstrated that Ephrin B3 binds to EphA3 and EphA5, two EphAs previously reported to be mutated in certain NSCLC cases [7] (Figure 3E). We also confirmed the reported interaction between Ephrin B3 and EphA4 [25] (Figure 3E). Ephrin B3 has been demonstrated to be a ligand of EphB3 and it was recently shown that in NSCLC cells overexpression of EphB3 may suppress metastatic signaling [11]. In order to examine if Ephrin B3 may act via EphB3 in our NSCLC cells we also analyzed interaction between Ephrin B3 and EphB3 by immunoprecipitation. However, we could not demonstrate Ephrin B3 to be a ligand of EphB3 in these cells (data not shown).

### Profiling of EphA2 complex in NSCLC reveals interaction with phospho-Akt Ser129 and p38MAPK

**Figure 4:**
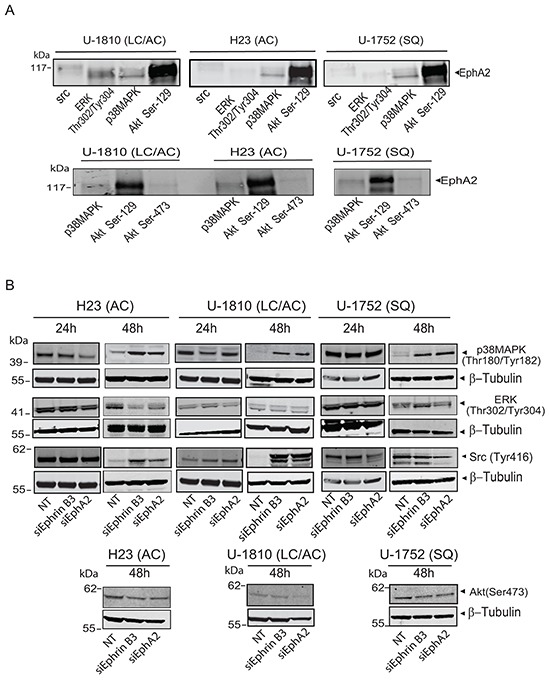
Ephrin B3 and EphA2 interact and control multiple proliferative kinases **A.** The interactions of EphA2 with total Src, phospho-ERK (Thr302/Tyr304), total p38MAPK, phospho-Akt (Ser129) and phospho-Akt (Ser473), were studied by immunoprecipitation in untreated U-1810, H23 and U-1752 NSCLC cell line. The resulting immunocomplexes were analyzed by western blot, with antibody against EphA2. **B.** H23 (AC), U-1810 (LC/AC) and U-1752 (SQ) cells were treated with Ephrin B3-, EphA2- or non-targeting (NT) siRNA. Indicated proteins were examined by western blot at 24 h or 48 h post siRNA. To control equal loading among the samples β-Tubulin was used.

To further study the Ephrin B3-EphA2 complex in terms of downstream signaling, we profiled for some of kinases involved in regulation of migration and proliferation i.e. Src, p38MAPK, phospho-ERK Thr302/Tyr304 and phospho-Akt Ser129 (Figure 4A, *top panel*). Interestingly, we found all these kinases to be in complex with EphA2 in LC/AC U-1810 cells (Figure 4A, *top panel*). Moreover, in the same cell lysate, Ephrin B3 was able to pull-down EphA2 (data not shown), indicating that when Ephrin B3 and EphA2 are in complex, phospho-Akt Ser129 is associating with EphA2 which is in contrast to reports on Ephrin A1/EphA2 complexes [15]. Interaction between phospho-Akt Ser129 or p38MAPK and EphA2 was also found in AC H23 and SQ U-1752 (Figure 4A, *top panel*). In contrast, Src and phospho-ERK immunoprecipitations did not reveal association to EphA2 in AC H23 and SQ U-1752 respectively, suggesting that the interaction with ERK and Src by EphA2 likely is cell type dependent (Figure 4A *top panel*). In contrast to our results on phospho-Akt Ser129 we did not reveal any interaction between phospho-Akt Ser473 and EphA2 in these NSCLC cells (Figure 4A, *bottom panel*). Hence these data show that Ephrin B3 binding to EphA2 results in another interaction with Akt Ser 129 than seen upon engagement with Ephrin A1.

Next we analyzed how a blockade in Ephrin B3 or EphA2 expression altered the phosphorylation of these growth factor receptor controlled kinases in NSCLC cells (Figure 4B). At 48h post siRNA transfection all three cell lines showed increased phosphorylation of p38MAPK (Figure 4B). At 24 h post siRNA no consistent pattern of ERK phosphorylation was evident whereas at 48h post siRNA AC H23 cells displayed decreased phospho-ERK levels after ablation of either Ephrin B3 or EphA2 expression while LC/AC U-1810 or SQ U-1752 did not (Figure 4B). At 24 h post siRNA no consistent regulation of phosphorylation of Src was evident whereas at 48 h post transfection increased Src Tyr416 phosphorylation was evident in AC H23 and LC/AC U-1810 cells but not in SQ U-1752 cells (Figure 4B). A slight decrease in phosphorylation of Akt Ser473 was observed in both AC H23 and SQ U-1752 cells upon inhibition of either Ephrin B3 or EphA2 and in LC/AC U-1810 cells after ablation of EphA2 expression (Figure 4B). Thus the downstream signaling effect upon Ephrin B3 or EphA2 blockade is cell type dependent.

### Ephrin B3, Ephrin A1 and EphA2 are concomitantly expressed in NSCLC clinical specimens

**Figure 5:**
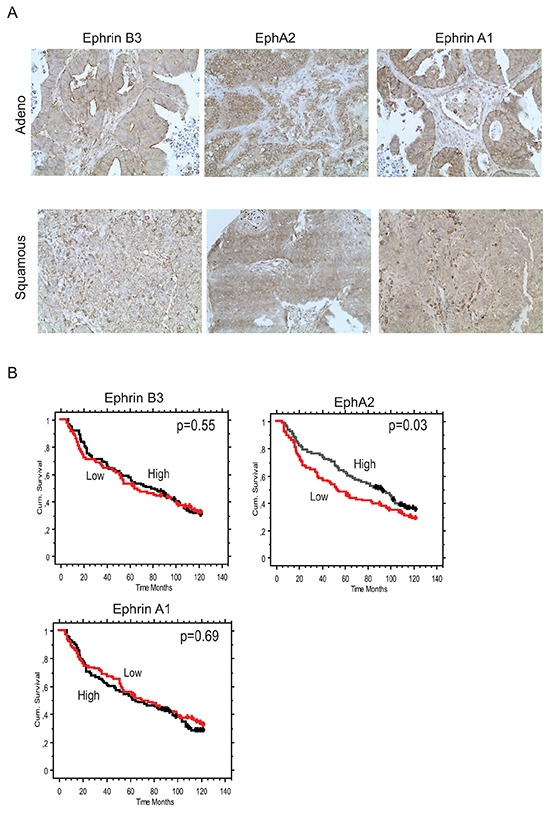
Concomitant Ephrin B3, Ephrin A1 and EphA2 expression in NSCLC clinical specimens Ephrin B3, Ephrin A1 and EphA2 expression levels and their relation to patient overall survival (OS) was analyzed in a cohort of Stage IA-Stage IB NSCLC specimen using immunohistochemistry. **A.** Examples of Ephrin B3, EphA2 and Ephrin A1 staining in adenocarcinoma and squamous cell carcinoma NSCLC specimens. 20x magnification. **B.** Kaplan-Maier curves showing the association between Ephrin B3, EphA2 and Ephrin A1 expression intensity levels and OS in the NSCLC patient cohort. Red line: Low score, black line: High score. Wilcoxon test was used for statistical assessment and the p-values are indicated.

An increased EphA2 expression has previously been reported in NSCLC [5, 6] and in some but not all studies (reviewed in [26]) been associated with poor overall survival in this tumor type . Recently Ephrin B3 mRNA expression was connected to increased risk of relapse in NSCLC [9] yet its protein expression levels have not been evaluated in NSCLC. Given our *in vitro* results, we analyzed as to what extent Ephrin B3, Ephrin A1 and EphA2 were expressed in early stage IA-IB NSCLC tumor specimens (Figure 5, Table 1, Supplementary Table S1).

**Table 1:** Ephrin B3, Ephrin A1 and EphA2 expression levels in NSCLC clinical specimens

	No. evaluable cases N^a^(%)	Non-Squamous Tumors N^a^(%)	Squamous tumors N^a^(%)
**Ephrin B3**	211	129	82
** Low**	99 (47)	51 (40)	48 (59)
** High**	112 (53)	78 (60)^b^	34 (41)^b^
**EphA2**	221	134	87
** Low**	114 (52)	63 (47)	51 (59)
** High**	107 (48)	71 (53)	36 (41)
**Ephrin A1**	221	134	87
** Low**	116 (52)	69 (51)	47 (54)
** High**	105 (48)	65 (49)	40 (46)

a number,

b 2-sided Fisher's Exact test, p=0.007

We found Ephrin B3, Ephrin A1 and EphA2 all to have higher expression in tumor tissue relative to surrounding non-tumorous tissue (Figure 5A). Co-presence of the three biomarkers in the same specimens was evident and Ephrin B3 had a significantly higher expression in both Ephrin A1 and EphA2 positive cases (Fisher's Exact test p-values = 0.0002 and 0.012, respectively), whereas the association between Ephrin A1 and EphA2 expression only showed a trend (p=0.05) (Table 1). In terms of associations with clinico-pathological characteristics, Ephrin B3 was significantly more expressed in non-squamous than in squamous tumors (Table 1), whereas EphA2 expression was higher in well-differentiated than in low-differentiated tumors (p=0.03). The prognostic role of tumor expression of Ephrin B3, EphA2 and Ephrin A1 in early stage NSCLC is presented in Figure 5B. Neither Ephrin B3 nor Ephrin A1 had an impact on survival in this cohort of NSCLC. On the other hand, EphA2 expression was found to be a positive prognostic factor. Median overall survival was 93 months vs 53 months is patients with high vs low EphA2 expression, respectively (p=0.037, Wilcoxon test) (Figure 5B).

## DISCUSSION

Tyrosine kinase signaling via EphA upon Ephrin A ligand binding activates and represses multiple cellular pathways in normal cells whereas in tumors these pathways are often altered e.g. by different Ephrin ligand expression or Eph binding pattern or by Eph kinase mutations resulting in increased proliferation-, migration- and invasion capacity [3]. This is evident also for EphA2 which is reported to drive tumor invasion and proliferation in NSCLC and other tumor types [5, 6, 12–17]. Here we for the first time show that blocking Ephrin B3 expression inhibits migration and invasion in NSCLC cells of different histology *in vitro.*

We present the novel finding that Ephrin B3 forms complex with EphA2 Ser897 in NSCLC of different histology and within such complex Akt Ser129 is found. This is in contrast to effects seen upon Ephrin A1 binding to EphA2 which has been reported to block EphA2 Ser897 and Akt Ser129, resulting in inhibition of migration/invasion of glioma and prostate cancer cells respectively [12, 15]. Moreover, our results which demonstrated prevention of NSCLC migration and invasion upon blockade of Ephrin B3 expression is in line with such a different function of Ephrin B3 on EphA2.

We previously found that inhibition of Ephrin B3 in NSCLC cells prevented Akt Ser129 phosphorylation and resulted in partial degradation of EphA2 [19]. If Ephrin B3 directly control Akt Ser129 phosphorylation levels or if ablation of Ephrin B3 expression results in EphA2 degradation and thereby Akt Ser129 down regulation remains to be explored. Miao et al. showed that multiple growth factors and their ligands i.e. HGF, bFGF, EGF and PDGF may control EphA2 Ser897 phosphorylation upon serum starvation [15]. Given that heterodimers of EphA2 and EGFR do exist in tumor cells [27], one may speculate that by altering the EphA2 interactome Ephrin B3 may influence such EphA2/EGFR heterodimers and in this way control Akt Ser 129 and EphA2 Ser897, resulting in block of EphA2 mediated invasion/migration signaling.

Down regulation of Ephrin A1 in breast cancer increases tumor invasiveness [28] illustrating that this may be a way to maintain EphA2 Ser897 phosphorylation. Given that we in NSCLC cells *in vitro* and in NSCLC patient material found a concomitant expression of Ephrin A1 and Ephrin B3, it is evident that NSCLC most likely uses another way to modulate EphA2 into a metastatic driver. From our data we speculate that Ephrin B3 binding to EphA2 involves another site of interaction than Ephrin A1 allowing EphA2 to stay active. Along this line, it was shown that co-expressed in the same tumor cell Ephrin A3 binding *in cis* (ligand and receptor on the same cell) can block EphA2 and EphA3 *in trans* (ligand and receptor on different cells) ligand binding [29]. Taken this into context of our results, one may hypothesize that Ephrin B3 and EphA2 also interact *in cis* and that this precludes Ephrin A1 to ablate EphA2 Ser897 phosphorylation when binding *in* *trans.*

We showed that the migration potential of NSCLC cells was clearly reduced when knocking Ephrin B3 *in vitro*. In contrast, our results suggest that the block in proliferation in response to such treatment is cell type dependent. As the cell lines express similar amount of Ephrin B3, Ephrin A1 and EphA2 we speculate that the differences in EphA2 interaction complex with the downstream signaling proteins p38MAPK, Src and phosphorylated ERK might be the underlying cause to this.

Residual cells, surviving Ephrin B3 or EphA2 blockade, displayed increased p38MAPK phosphorylation. As we previously found p38MAPK to be in complex with IGF-1R and to be a pro-survival factor in NSCLC cells [30] our results may also indicate that crosstalk exists between EphA2 and IGF-1R similarly as reported for EphA2/EGFR [27] allowing certain EphA2 signaling and proliferation capacity to be maintained. Hence it would be interesting to combine Ephrin B3 blockade with either EGFR or IGF-1R signaling inhibiting strategies in order to further prevent proliferation of NSCLC cells. Such a therapy approach is also highly relevant for EGFR-mutated NSCLC given that a high EphA2 signaling was recently shown to impart on erlotinib sensitivity [31].

Apart from EphA2, also EphA4 [9] and EphA5 [10] play roles in NSCLC migration and proliferation respectively. Thus EphA4 overexpression was shown to block NSCLC migration [9] while blocking EphA5 reduced proliferation and sensitized NSCLC cells to RT [10]. We present the novel finding that Ephrin B3 also is a ligand of both EphA4 and EphA5 alongside EphA3. Despite therelatively low expression of these Ephs in the NSCLC cells examined compared to EphA2, Ephrin B3 may still act via these to exert some of its effect and thus blocking Ephrin B3 could also potentially target these Ephs. Our data on Ephrin B3-EphA4 interaction and our preliminary data which suggests that blocking either EphA4 *per se* or Ephrin B3 in combination with EphA4 in fact cause cell death in certain NSCLC cells (Novak et al., unpublished data) is contrast to Saintigny et al., who found EphA4 blockade to inhibit migration [9]. Taken our results and given a report on embryonic kidney HEK293 cells, where Ephrin B3 was required to prevent caspase-mediated EphA4 cleavage and subsequent apoptosis, we speculate that Ephrin B3 may exert such a function on EphA4 in NSCLC cells [25]. However, our analyses of EphA4 cleavage after Ephrin B3 blockade did not reveal any caspase-mediated fragments of EphA4 in these NSCLC cells (data not shown). Hence our results suggest that other mechanisms contribute to the Ephrin B3 mediated effects with respect to EphA4 at least in these NSCLC cells.

Ephrin and Eph signaling in tumors are reported to control EMT signaling [23]. Thus lack of E-cadherin expression in breast cancer cells caused improper localization of EphA2 to membrane ruffles, rather than to the cell-cell conjunctions and resulted in a metastatic phenotype [24]. Moreover, in glioma cells Ephrin B3 was shown to interact with the EMT signaling component Rac and in this way control its activation and glioma cell invasion capacity [20]. In our study we found maintained or increased E-cadherin expression in Ephrin B3- ablated NSCLC cells. One may speculate that such E-cadherin will allow for the restoration of EphA2 signaling at cell-to cell junctions and thereby blockade of migration. Further studies on E-cadherin localization alongside EphA2 in NSCLC cells are yet required. Albeit Nakada et al., showed Ephrin B3 and Rac interaction [20] we did not found any alteration of Rac expression upon blockade of Ephrin B3 in NSCLC cells. Hence further analyses of Ephrin B3:s direct association with EMT signaling in NSCLC is warranted.

In our analysis of stage IA-IB NSCLC tumors, Ephrin B3 was expressed concomitantly with EphA2 and Ephrin A1 indicating that Ephrin B3 may *in vivo* also have capacity to act on EphA2. Moreover, we observed a higher degree of Ephrin B3 expression in non-squamous than in squamous tumors. Our analyses did not reveal any association between Ephrin B3 expression and patient overall survival, whereas a high EphA2 expression was associated with improved survival in our NSCLC clinical cohort. Both biological and methodological aspects may contribute to the lack of connection between high Ephrin B3 expression and poor overall survival of the NSCLC patients. First, we used TMA in which the tumor cores were taken from solid tumor tissue hence we cannot rule out that our results are influenced by heterogenity in Ephrin B3 expression which we do not capture in TMA. It would therefore be interesting to further analyze Ephrin B3 in whole tumor sections especially at the invasive front of tumors. Second, given *in vitro* data from us and others showing the importance of Eph receptors expression concomitantly with Ephrin B3, it might well be so that the summarized expression levels of all Ephs in a certain tumor section will dictate the importance of Ephrin B3 as a biomarker of survival not Ephrin B3 expression level *per se*. Hence profiling of ratios between EphA2, EphA3, EphA4 and EphA5 expression in the same specimen or their interaction with Ephrin B3 would be interesting to further explore in relation to NSCLC patient survival, especially in non-squamous NSCLC cases. As we found EphA2 Ser 897 to be in complex with Ephrin B3 in NSCLC cells *in vitro* and given that such phosphorylation also was reported in NSCLC *in vivo* in context of EGFR-mutation [31], we think that further analyses of NSCLC material with respect to this interaction should be pursued. With respect to EphA2, our results are contradictory to those reported by Brannan et al., who found high EphA2 to be associated with short recurrence time and development of metastasis in NSCLC [6] but also that a high EphA2 expression within tumor conferred short progression-free and overall survival time [5]. One may speculate that difference in tumor stage of the included cases of our study vs Brannan et al., could contribute. Thus our cohort consisted only of stage IA-IB NSCLC whereas their study contained about 25% of stage III and IV tumors [6].

In conclusion, we identified Ephrin B3 as an interaction partner of EphA2, EphA3, EphA4 and EphA5 in NSCLC cells. Our study shows that migration and invasion capacity is blocked in NSCLC cells of different histology when Ephrin B3 expression is impeded, while proliferation is reduced in a cell type dependent manner. In the examined early stage NSCLC cohort we did not found any correlation of Ephrin B3 expression *per se* and patient overall survival yet we found Ephrin B3 to have a higher expression in NSCLC specimen alongside EphA2. Thus our results support that at least for a subset of NSCLC tumors blocking Ephrin B3 and EphA2 signaling may constitute a novel therapeutic avenue.

## MATERIALS AND METHODS

### NSCLC cell lines

The adenocarcinomas H23, H1299, A549, H661, H157; adenosquamous H125 (purchased from American Type Culture Collection (ATCC, Manassas, VA, USA)); CL1-5 (kind gift from Dr Yang Pan-Chyr (Institute of Biomedical Sciences, Academina Sinica, Taiwan) [21, 22]), mixed phenotype large cell/adenocarcinoma U-1810 and squamous U-1752 cells (given from Uppsala University [32, 33] were used. Cells were maintained in RPMI-1640 medium supplemented with 10% heat-inactivated fetal bovine serum (FBS) and 2mM L-glutamine (both from Sigma Aldrich, Stockholm, Sweden).

### RNA interference of Ephrin B3 or EphA2

siRNA targeting a previously reported unique Ephrin B3 sequence 5′-CCAGGAGTATAGCCCTAAT-3′ [20] was purchased from Qiagen (Maryland USA). For EphA2 siRNA experiments the On-Target plus Smart pool which is a mixture of four siRNAs against EphA2 (5′-UGAAUGACAUGCCGAUCUA-3′, 5′-GA AGUUCACUACCGAGAUC-3′, 5′-CAAGUUCGUUGA CAUCGUC-3′, 5′-UCACACACCCGUAUGGCAA-3′) (Thermo Fisher Scientific Inc, IL, USA) was used. As non targeting (NT) siRNA the On-Target plus Non-targeting pool was applied (Thermo Fisher Scientific Inc). All transfections were carried out with 100 nM of Ephrin B3, EphA2 or NT siRNA using Dharmafect 1 transfection reagent (Thermo Fisher Scientific).

### Quantitative real time PCR analysis

RNA knock-down of Ephrin B3 and EphA2 after siRNA transfection was confirmed by real-time quantitative PCR (RT-Q PCR). Qiagen RNeasy kit (Qiagen, Sollentuna, Sweden) was used to extract total RNA and 1 μg RNA was reversed transcribed to cDNA using 2.5 μM random hexamer primers, 2 mM dNTPs, 5.5 mM MgCl, 8 U RNAse Inhibitor, 25 U MultiScribe Reverse Transcriptase in reverse transcription buffer (Applied Biosystems, Stockholm, Sweden) with heating for 25°C, 10 min, 37°C, 1 h and extention at 95°C for 5 min.

Expression levels of Ephrin B3 or EphA2 were analyzed using 1 μl of cDNA mixed with the TaqMan® Fast Advanced Master Mix and Gene Expression Assay mix for Ephrin B3 (ID:Hs00154861_m1) or EphA2 (ID:Hs00171656_m1))(Applied Biosystems). To control for loading difference the glyceraldehyde-3-phosphate dehydrogenase (GAPDH, ID:Hs02758991_g1) was applied. The ABI Prism 7900HT Sequence detection system (Applied Biosystems) was used in these analyses with the following running conditions: 95°C, 20 s, 45 cycles of 95°C, 1 s and 60°C, 20 s. Relative expression values were calculated using the 2^−ΔCt^ method.

### Assessment of proliferation, migration and invasion potential

The proliferation and migration experiments in H23, U-1810 and U-1752 cells were carried out by transfecting 8*10^5^ cells in 10 cm dishes with 100 nM of the indicated siRNA for 24 h in serum free transfection RPMI-1640 media. Subsequently media containing 10% serum was added for 24 h to 48 h followed by starvation in serum free media for another 24 h to 48 h. siRNA knockdown of expression was analyzed by RT-Q PCR. Proliferation was assessed at 24 h to 48 h post siRNA removal, by counting viable cells in Bürker chamber using trypan blue staining. Values given in Ephrin B3 or EphA2 transfected samples are given relative to NT siRNA. Means± SD from three separate transfections is shown.

To assess migration potential, transwell assay was used. At 24 h post siRNA transfection and following 24 h recovery in media containing 10% serum followed by serum starvation for an additional 24 h cell migration potential was assessed. The serum starved cells were seeded in the upper chamber of the transwell insert (Transwell: Millipore, cat.no PIEP15R48, MA). The migration capacity of the cells over the membrane towards the bottom chamber in which serum-containing RPMI-1640 media was added was examined after 20 h. Cells on the membrane were fixed in 2.5% glutaraldehyde solution and visualized by staining with 0.5% crystal violet solution. The number of cells was quantified by light microscope and the values given in Ephrin B3 or EphA2 siRNA transfected cells are relative to NT siRNA. For CL1-5 cells transfection was carried out as above but with a 48 h transfection time followed by 24 h with media containing serum and another 24h in serum free media. Proliferation of Ephrin B3-, EphA2- or non-targeting siRNA transfected CL1-5 was examined as above. To assess invasion potential of the serum-starved CL1-5 cells, cells were seeded in the upper chamber of the transwell insert which was covered with growth factor-reduced matrigel (2 μg, Becton Dickinson) and after 20 h cells on the bottom membrane were fixed, stained and the total amount of cells counted. Values given for Ephrin B3 or EphA2 siRNA transfected cells with respect to proliferation or invasion are given relative to NT siRNA. Data from two separate transfections are shown.

### Immunoblotting

Protein were extracted from cells by resuspension in RIPA buffer (50 mM Tris-HCl (pH 7.4), 150 mM NaCl, 0.5% Igepal, 5 mM EDTA (pH 8.0), 0.1% SDS supplemented with proteases inhibitors (Roche, Mannheim, Germany) followed by sonication and centrifugation (10,000 g/10 min) to clear out insoluble cell material. The bicinchoninic acid (BCA) assay (Interchim, MontiuconCedex, France) was used to quantify protein concentration. Proteins (50 μg of total cell lysates) were separated on either 4-12% Bis-Tris or 3-8% Tris-Acetate NuPAGE® gels (Invitrogen AB, Stockholm, Sweden). Proteins were subsequently blotted from the gels to nitrocellulose membrane (Hybond-C Extra, Amersham Biosciences) and blocked in Odyssey® blocking buffer TBST 1:1. Antibodies were added at + 4°C for 16h: Anti-Ephrin B3 (ab101699; 1:500), Anti-Ephrin A1 (ab 65072; 1:300) (both from Abcam, Cambridge Science Park, Cambridge, UK), Anti-EphA2 (#34-7400, 1:500; Invitrogen), Anti-phospho-Akt Ser473 (#9271, 1:1500), Anti-phospho-p44/42 MAP Kinase Thr202/Thr204 ERK (#9101, 1:500), Anti-phospho-p38 MAP Kinase Thr180/182 (#9211, 1:500), Anti-phospho Src Tyr416 (#2101, 1:500), Anti-vimentin (#5741, 1:1000) and Anti-Rac (#2465P, 1:300) (all from Cell Signaling MA, USA). Anti-E-cadherin antibody (#610181, 1:1000) was from BD Biosciences (MD, USA). Antibodies for EphA4 (#sc-921, 1:100), EphA3 (#sc-920, 1:500) and EphA5 (#sc-1014, 1:500) were all from Santa Cruz Biotechnology (TX; USA).

To control for loading differences, β-Tubulin (#T7816, 1:5000, Sigma Aldrich) was used. As secondary antibodies, anti-rabbit or anti-mouse antibodies (#5151 or #4408, 1:15000) from LI-COR Biosciences (Bad Homburg, Germany) were applied for 1 h. The resulting protein expression was examined and recorded by the use of the Odyssey® Sa Infrared Imaging System (LI-COR Biosciences).

### Immunoprecipitation

Immunoprecipitation was carried out from 800 μg total cell lysate in 100 μl. Lysis of NSCLC cells were carried out in buffer (25 mM Tris-HCl pH 7.4, 150 mM NaCl, 1 mM EDTA, 1% Nonidet P-40 and 5% glycerol) for 15 min on ice. 5 μg of Ephrin B3, EphA2, phospho-Akt Ser473 antibodies (as above) or total Anti-Src (#2123), total p38 MAP Kinase (#9212S) USA) or phospho-Akt Ser129 (#13461S) antibodies (all from Cell Signaling) were added to cell lysate for 1h in which IgG (# 12370, Millipore) serve as a negative control. To fish out the immunoprecipitation conjugates protein G-Sepharose beads (Millipore) were added to the samples for 1 h at 4°C. Beads were after rinsing in lysis buffer (Thermo Fisher Scientific) eluted for bound antigens using sample buffer. Separate immunoprecipitations were analyzed using immunoblotting with antibodies towards EphA2, EphA4, EphA3, EphA5 and EphB3 with antibodies given under immunoblotting section. In addition phospho-EphA2-Ser897 (AP3722a, 1:500, Abgent, CA, USA) was used.

### Proximity ligation assay (PLA)

To detect interactions between Ephrin B3 and EphA2 proximity ligation assay (PLA) was applied. NSCLC cells were seeded onto slides and after 24 h of growth fixed by incubating the slides in 4 % paraformaldehyde solution follow by washing in PBS and dehydration in 70 %, 85 % and 100 % ethanol. The slides were subsequently blocked for 1 h in 5 % BSA with 0.1 % Triton X-100. Antibodies towards Ephrin B3 (ab101699, Abcam) and EphA2 (374400, Life technologies) were applied for 1h at dilution 1:350. To visualize the protein interactions the antibody probed slides were incubated for 1 h with two different PLA probes (provided with the Duolink II assay kit (OlinkBioscience, Uppsala, Sweden)), which are directed against either antibody at 1:5 dilution in antibody diluent. The ligation and amplification stocks were added according to the manufacturer's protocol. To visualize cell nuclei DAPI was added to the mounting medium. The PLA staining were analyzed using an Axioplan 2 (Zeiss) fluorescent microscope with emission filters for DAPI and Texas Red. Pictures were takne at 20x mangification using a CCD camera (Hamamatsu). The positive interactions between the two targets are indicated with red fluorescent dots. As a control, sample without the primary antibodies were used.

### Human NSCLC tissue microarray (TMA)

From a clinical material of 228 NSCLC cases, 225 specimens contained sufficient material to be evaluated for Ephrin B3, EphA2 or Ephrin A1 expression. The clinical and pathological characteristics are given in Supplementary Table S1. Briefly, the patient cohort consisted of 225 subjects with node-negative stage I NSCLC who underwent curative surgery at the Dept. of Cardiothoracic Surgery, Karolinska Hospital, Stockholm, Sweden, between 1988 and 2002. From the formalin-fixed and paraffin embedded NSCLC specimens a trained pathologist constructed a tissue microarray (TMA) by selecting two representative tumor tissue rich biopsies from each sample into a recipient TMA block. The quality and representability of the selected sections with respect to histology was verified and subsequently the TMA slide was stained for Ephrin B3, EphA2 or Ephrin A1 protein expression. Prior to antibody addition xylene was used to deparaffinize the tissue followed by rehydratation in ethanol and deionized water and retrieval of antibody epitopes by immersing in sodium citrate buffer pH6 and incubation in 0.5% hydrogen peroxide solution. The following primary antibodies were used: Ephrin B3 (ab101699; 1:500), Ephrin A1 (ab65072; 1:500) both from Abcam, Cambridge Science Park, Cambridge, UK and EphA2 (#34-7400, 1:200; Invitrogen). To reveal primary antibody staining, a biotinylated anti-rabbit IgG secondary antibody (Vector Labs) was added (30 min, 1:200) with followed by incubation for 30 minutes with avidin-biotin peroxidase complex solution and followed by development of staining by adding a 3,3-diaminobenzidine solution for at least 5 min. IHC stainings were evaluated by a trained pathologist who was blinded to the clinical outcomes. To each case, a semi quantitative score was assigned based on the product of staining intensity (from 0 to 3, corresponding to no staining, low, moderate and strong expression, respectively) and the percentage of positive tumor cells (1 = <25%; 2 =<50% ; 3 =<75%; 4 >75%). In order to be able to categorize each biomarker expression into two numerically comparable groups as either “Low” or “High”, Ephrin A1 was classified as low if scored 0 to 6 and high if scored 8 to 12. The corresponding values for Ephrin B3 were 0-4 (low) and 6-12 (high) and for EphA2 they were 0-2 (low) and 4-12 (high). The association between Ephrin B3, EphA2 or Ephrin A1 expression and clinical parameters was analyzed with a 2-sided Fisher's Exact test. For the purpose of association analyses by histology, tumors were further classified as Squamous (including squamous-cell and adenosquamous carcinoma) and Non-Squamous (including adenocarcinoma, large cell and NOS cases). Overall survival was recorded as time between surgical resection to time of death (by any cause) or date of last follow up. Median follow up in 74 living patients was >10 years (IQR 117->122 months). Eight cases were excluded from survival analysis because of peri-operative mortality (n=3), administration of post-operative chemo- or radiotherapy (n=3) and R1 resection. The Kaplan–Meier method was applied to reveal effects of the biomarkers on survival in which Wilcoxon test was used for statistical assessment. The study was approved by the Karolinska Institutet ethical committee (2005/588-31/4).

## SUPPLEMENTARY FIGURES AND TABLE


