# Supplementary material for: Ephrin B3 interacts with multiple EphA receptors and drives migration and invasion in non-small cell lung cancer
Source: Oncotarget. 2016 Aug 11;7(37):60332–47. doi: 10.18632/oncotarget.11219 (PMC5312387; doi:10.18632/oncotarget.11219)
Supplement: Supplementary file 1 [file oncotarget-07-60332-s001.pdf]

Ephrin B3 interacts with multiple EphA receptors and drives migration and invasion in non-small cell lung cancer

SUPPLEMENTARY FIGURES AND TABLE

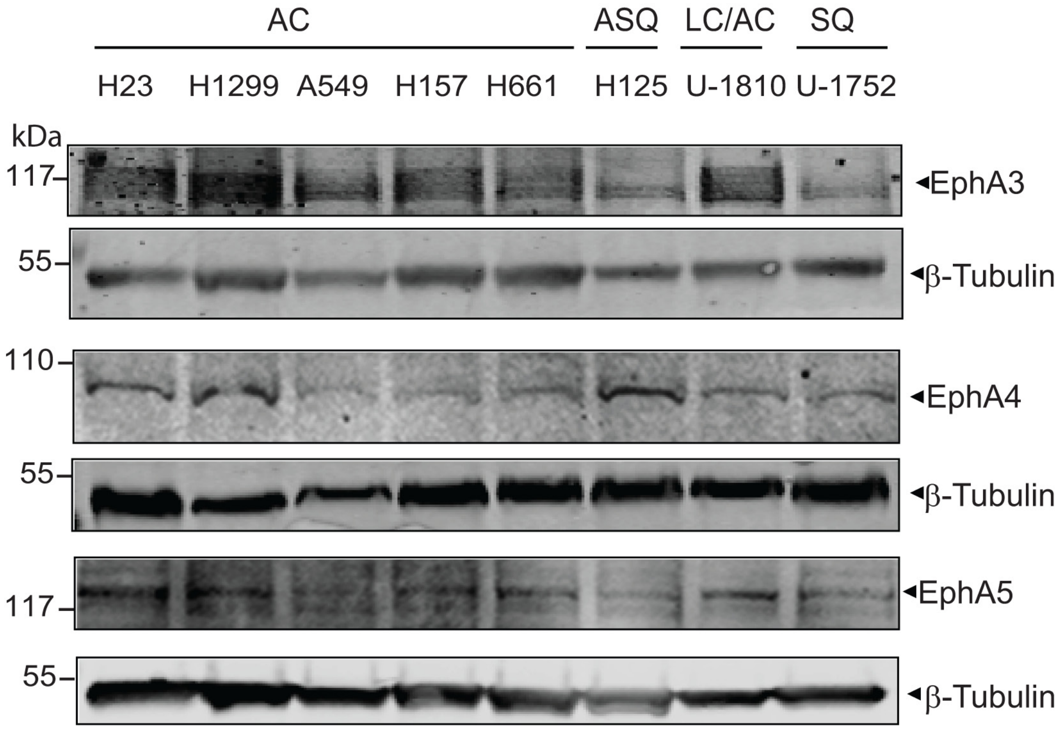

Supplementary Figure S1: Expression of EphA3, EphA4 and EphA5 in NSCLC of different histologies. NSCLC cell lines with the histology adenocarcinoma (AC), adenosquamous carcinoma (ASQ), mixed phenotype (large cell/adenocarcinoma, LC/AC) or squamous (SQ) were profiled for the expression of EphA3, EphA4 and EphA5, using western blotting. To control equal loading among the samples, β-Tubulin was used.

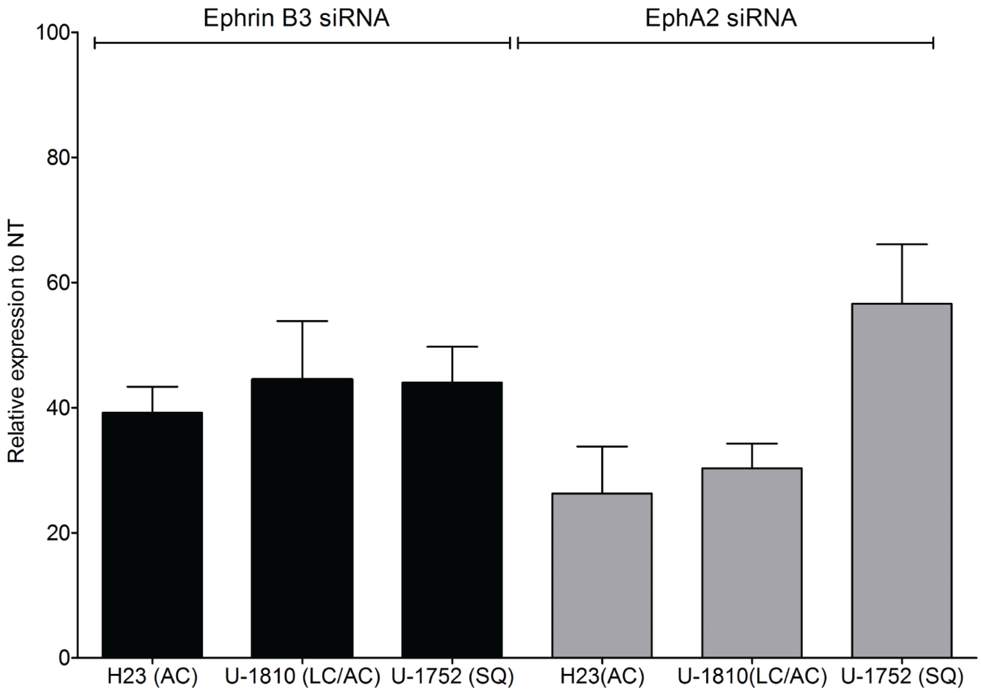

Supplementary Figure S2: Down regulation of Ephrin B3 and EphA2 expressions in NSCLC cells. H23 (AC), U-1810 (LC/AC) or U-1752 (SQ) cells were transfected with Ephrin B3, EphA2 or non-targeting (NT) siRNA as indicated in Figure 1. Reduced Ephrin B3 and EphA2 expressions in all cell lines were confirmed by real time quantitative PCR, where their expression levels were first related to GAPDH housekeeping gene and then calculated as % of NT, set to 100%. Ephrin B3 siRNA: black bars; EphA2 siRNA: grey bars.

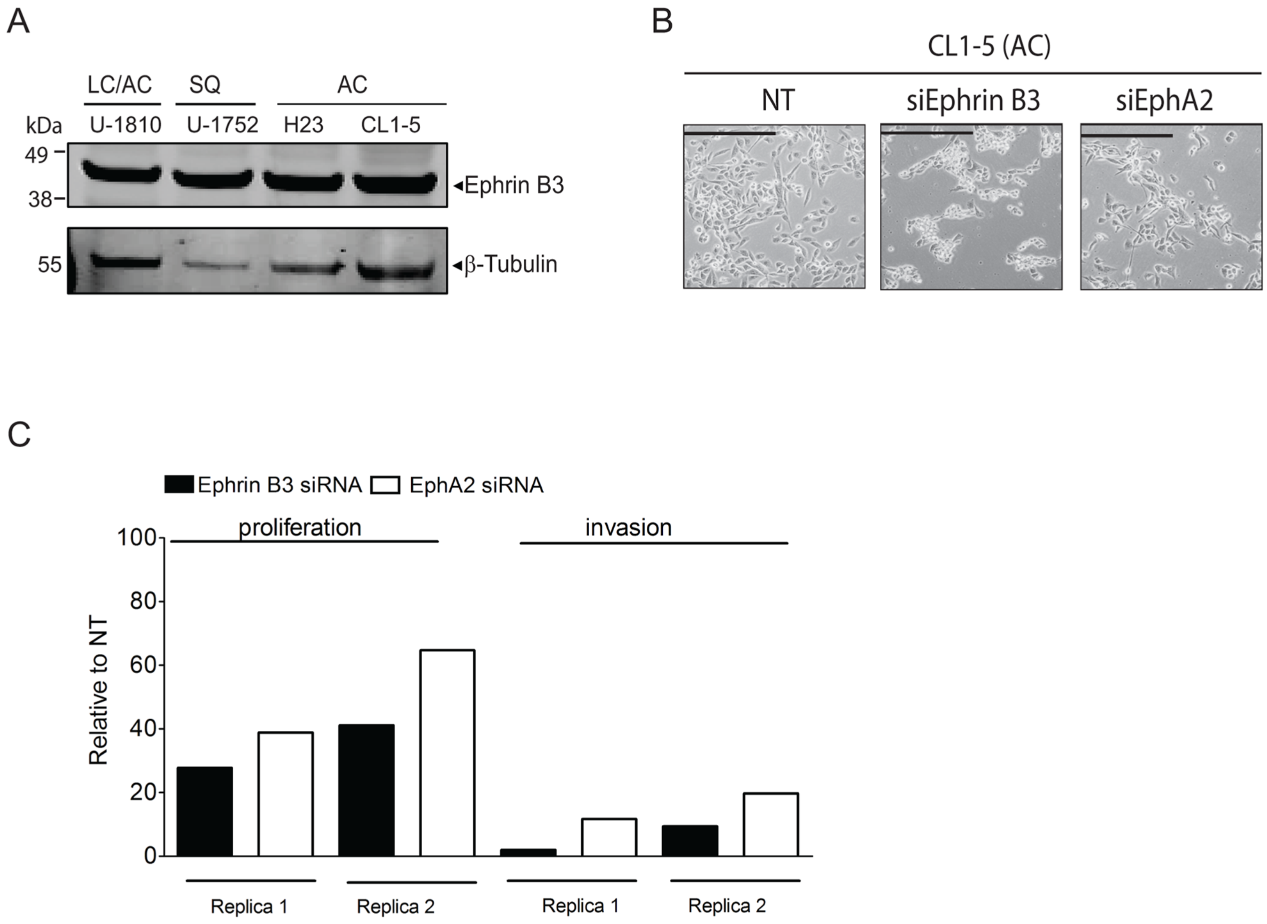

Supplementary Figure S3: Endogenous expression of Ephrin B3 or EphA2 drives invasion of NSCLC cells. A. Ephrin B3 expression was examined in NSCLC CL1-5 cells by western blotting and with β-Tubulin as loading control. The other NSCLC cell lines used in migration analyses (Figure 1) are shown for comparison. **B.** Photos showing effect on cell morphology by Ephrin B3 or EphA2 siRNA in CL1-5 cells. **C.** Assessment of proliferation capacity in CL1-5 cells quantified as number of living cells by Trypan blue. Data is presented as % of NT treated cells. Invasion capacity was examined in matrix gel coated transwell assay in which the total number of cells was scored as indicated in Figure 1D. The percentage of migrating cells relative to NT is given. Data presented is from two biological replicates.

Supplementary Table S1: Clinico-pathological characteristics of NSCLC patients

		N (%) 225 (100)	Non-Squamous N (%) 138 (61)	Squamous N (%) 87 (39)
Age, median (IQR)	years	69 (62-74)	67 (59-73)	71 (66-75)
Gender	Male	119 (53)	59 (43)	60 (69)
	Female	106 (47)	79 (57)	27 (31)
Stage at diagnosis	IA	112 (50)	80 (58)	32 (37)
	IB	113 (50)	58 (42)	55 (63)
Grading (G)	G1	54 (24)	48 (35)	6 (7)
	G2	85 (38)	42 (30)	43 (49)
	G3	82 (36)	44 (32)	38 (44)
	NR	4 (2)	4 (3)	
Smoking history	Never	19 (8)	18 (13)	1 (1)
	Former	65 (29)	40 (29)	25 (29)
	Current	116 (52)	65 (47)	51 (59)
	NR	25 (11)	15 (11)	10 (11)
Histology	Adenocarcinoma	126 (56)		
	Large Cell/NOS	12 (5)		
	Squamous-cell carcinoma	65 (29)		
	Adenosquamous	22 (10)		

Abbreviations: N, number; G, Differentiation grade (G1=well; G2= moderate; G3=low differentiation); NR, not reported.
